# Supplementary material for: Exome sequencing of bulked segregants identified a novel TaMKK3-A allele linked to the wheat ERA8 ABA-hypersensitive germination phenotype
Source: Theor Appl Genet. 2020 Jan 28;133(3):719–36. doi: 10.1007/s00122-019-03503-0 (PMC7021667; doi:10.1007/s00122-019-03503-0)
Supplement: Supplementary file 1 — Fig. S1— Crossing strategy to generate the ERA8 backcross mapping population. Fig. S2—WT and ERA8 parental seed germination assay. Fig. S3—Insertions or deletions between WT and ERA8. Fig. S4—ABA response to number of QTL in the Louise/ZakERA8 RILs. Fig. S5—QTL analysis of Louise/ZakERA8 heading date and height. Fig. S6—WT and ERA8 coding sequence of TaMKK3-A (PDF 629 kb) [file 122_2019_3503_MOESM1_ESM.pdf]

SUPPLEMENTAL MATERIAL 1: FIGURE S1 – S6

Martinez SA, Shorinola O, Conselman S, See D, Skinner DZ, Uauy C, and Steber CM (2020) Exome sequencing of bulked segregants identified a novel *TaMKK3-A* allele linked to the wheat *ERA8* ABA-hypersensitive germination phenotype. Theor Appl Genet

Correspondence: Camille M. Steber
email: camille.steber@usda.gov
phone: (509) 335-2887

Fig. S1 - Crossing strategy to generate the *ERA8* backcross mapping population
Fig. S2 - WT and *ERA8* parental seed germination assay
Fig. S3 - Insertions or deletions between WT and *ERA8*
Fig. S4 - ABA response to number of QTL in the Louise/Zak*ERA8* RILs
Fig. S5 - QTL analysis of Louise/Zak*ERA8* heading date and height
Fig. S6 - WT and *ERA8* coding sequence of *TaMKK3-A*

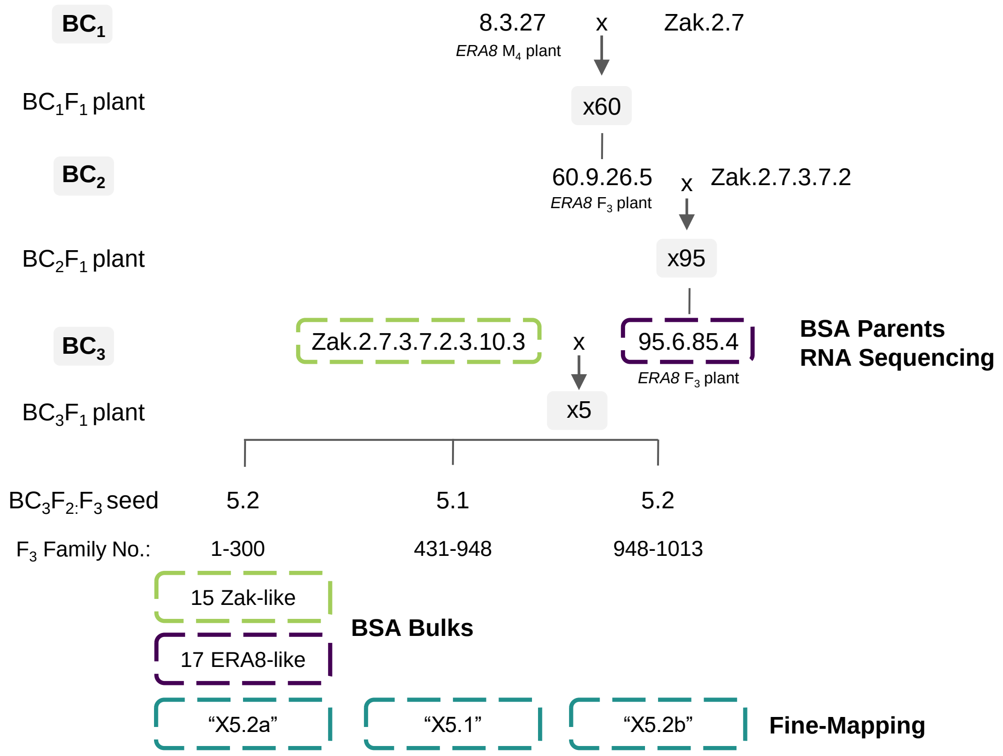

Fig. S1 Crossing strategy to generate the *ERA8* backcross mapping population. Zak WT parent (Zak2.7.3.7.2.3.10.3), the *ERA8* parent (95.6.85.4), *ERA8*-like bulk, and Zak-like bulk samples indicated by the dashed **green** and **purple** boxes were used in the bulked segregant analysis (BSA) exome sequencing. The BC_3F_2:F_3 lines used in the fine mapping study are indicated by the dashed **blue** boxes. The parent on the left of the ‘x’ is the female plant used in the cross.

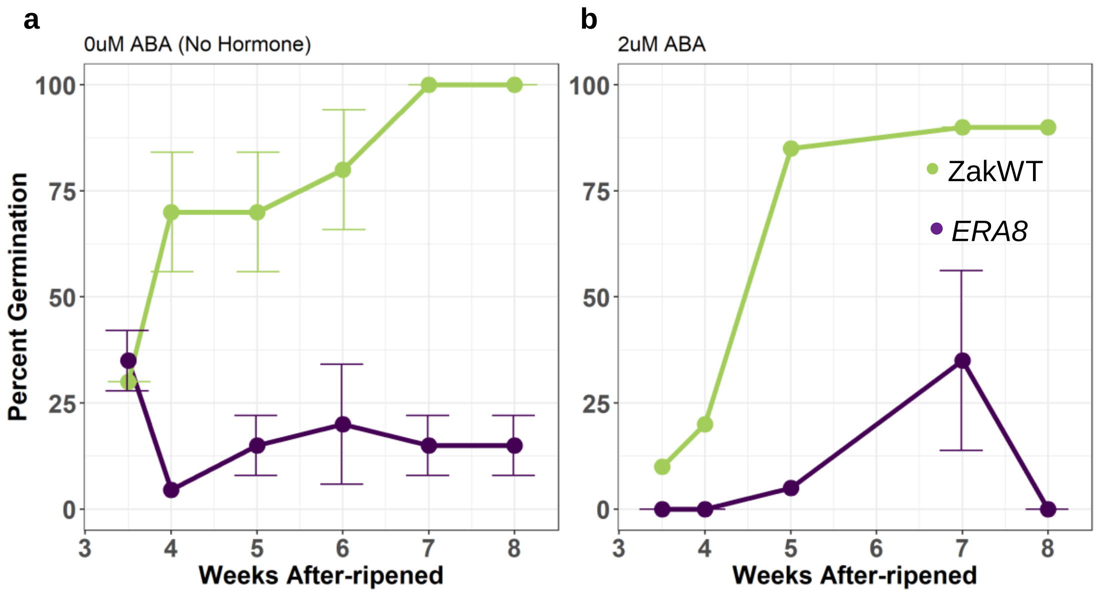

Fig. S2 WT and *ERA8* parental seed germination assay. An after-ripening time course of the *ERA8* (purple) and ZakWT (green) parents from the Zak/Zak*ERA8* was conducted without ABA (No Hormone; **a**) and with 2 μM ABA (**b**). Percent germination after 5 days of imbibition at 30°C was calculated from germination assays of 20 whole seeds each. The five week after-ripened time point was chosen for bulk segregant analysis.

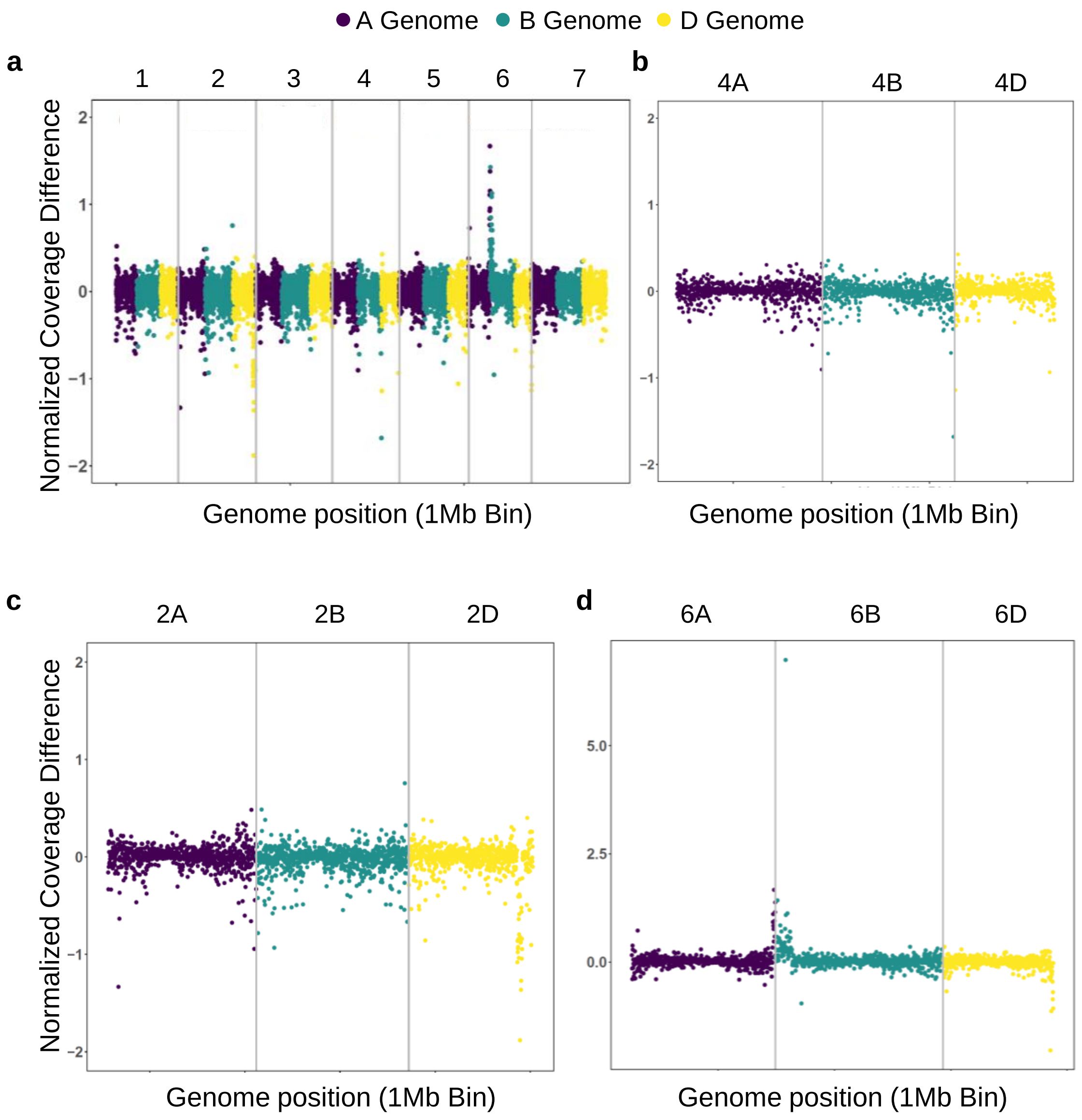

Fig. S3 Insertions or deletions between WT and *ERA8*. To identify potential insertions and deletions, read coverages in Zak WT were compared to *ERA8* across the: **a**) whole genome; **b**) group 4 chromosomes; **c**) group 2 chromosomes, and **d**) group 6 chromosomes. Insertions and deletions in *ERA8* are indicated by a positive or negative difference, respectively. The A genome is shown in purple, the B genome in green, and the D genome in yellow.

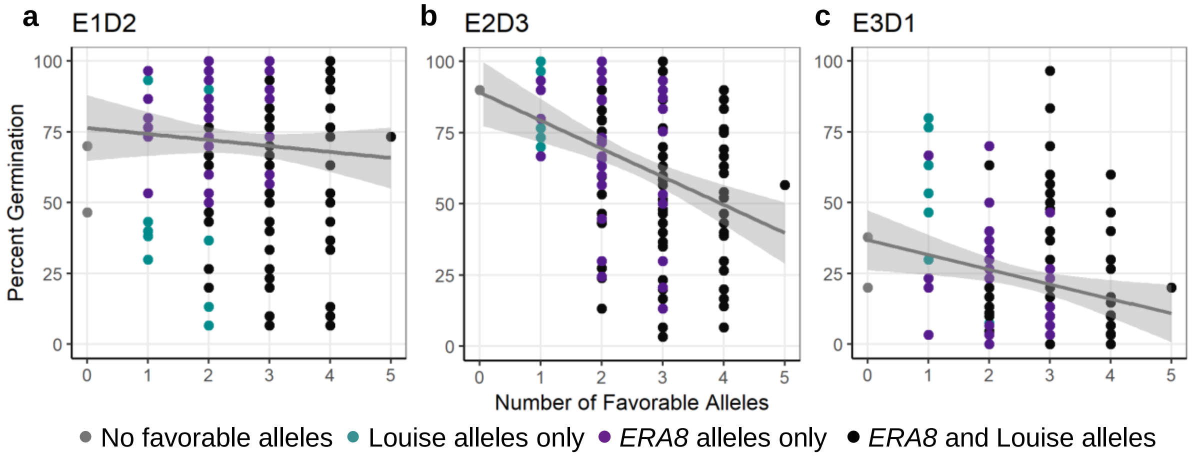

Fig. S4 ABA response to number of QTL in the Louise/Zak*ERA8* RILs. The number of favorable alleles that increase ABA sensitivity is compared against percent germination. Environments E1, E2, and E3 are shown after **a**) two, **b**) three, and **c**) one day of imbibition, respectively. Individual RILs are shown to have no tolerant alleles (grey dots), only the Louise (blue) or *ERA8* (purple) parent contributes to the tolerant allele, or both parents (black) contribute to the tolerant alleles. A simple linear regression line is shown using `geom_smooth(method = lm)` in the ggplot R package.

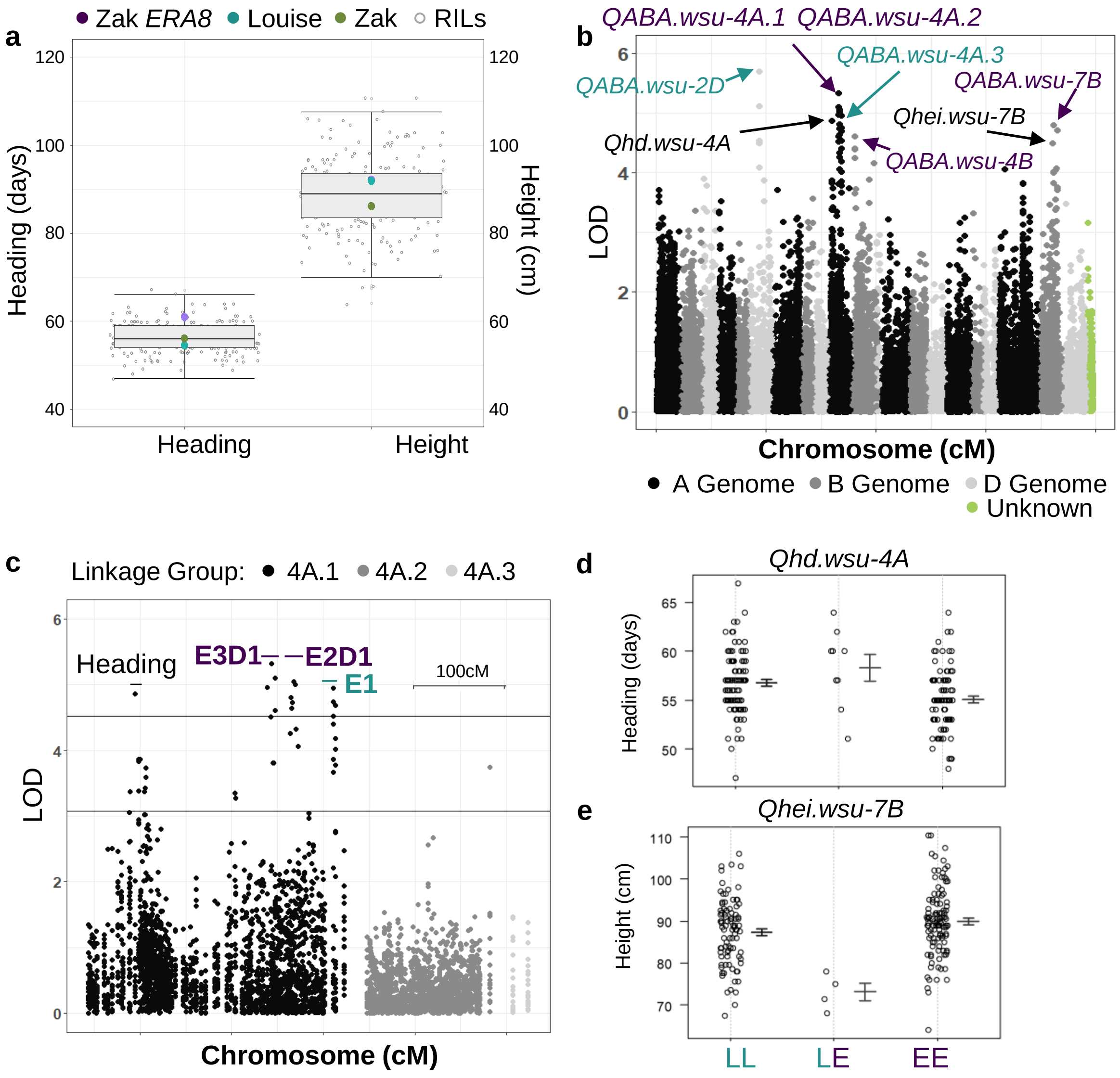

Fig. S5 QTL analysis of heading and height data. LOD scores for **a**) heading and height of the Louise/Zak*ERA8* RIL population are shown across **b**) all chromosomes (cM) and **c**) only for chromosome 4A. are shown. Significant QTL for height (*Qhei.wsu;* black), days to heading (*Qhd.wsu;* black), and ABA sensitivity (*QABA.wsu*), with a threshold of $p <$ 0.10 are shown. QTL where *ERA8* or Louise contributes to the ABA sensitivity are shown as purple or blue, respectively. Alleles were compared between heading date (**d**; days) and height (**e;** cm) differences in the RIL population separated by Louise (LL) and *ERA8* (EE) alleles. Note that height was taken from a single plant and not multiple plants across a plot.

a ■ TaMKK3-A C660A mutation ■ EMS G1093A mutation

Zak WT

```
ATGTCGGGGCTAGAGGAGTTGAAGAAGAAGCTGCAGCCCTTGCTGTTCGACGACACGGACAAGGGCGGCGTCAGTACCCGGGTTCCCTTCCCGGAGGATACATGCGATTCCTATGT
GGTGTCTGATGGTGGAACGATAAATTTACTGAGTAGATCGTTTGGTGAGTATAACATCAATGAGCATGGCTTTCATAAGCGAAGCACCGGACCAGAAGAGCCAGATAGCGGTGAGA
AGGCATACCGATGTGCCTCTGAAGACATGCACATATTTGGTCCCATTGGGAATGGAGCAAGCAGTGTCGTGCAGAGAGCTATTTTCATACCAGTTCATCGAATTCTGGCCTTGAAG
AAGATAAACATATTTGAGAAGGAGAAAAGGCAACAAATTCTTAATGAGATGAGAACATTATGTGAAGCAAGTTGCTATCCTGGTTTAGTTGAATTCCAGGGTGCATTTTACATGCC
TGATTCTGGACAAATAAGCATTGCCCTTGAATACATGGATGGTGGCTCTTTAGCAGACGTTATAAAAGTCAAGAAATCAATACCAGAGCCAGTTCTTGCACATATGCTACAGAAAG
TGTTACTTGGCCTGAAGTACTTGCATGAAGTAAGACATCTAGTGCATAGAGATCTAAAGCCAGCAAATTTACTGGTAAAACTTAAGGGCGAAGCAAAAATTACAGATTTTGGTGTA
AGTGCTGGTTTGGACAATACAATGGCTATGTGTGCTACCTTTGTAGGCACTGTGACATATATGTCACCTGAGAGAATCCGCAATGAGAACTACTCCTATGCTGCTGATATTTGGAG
TCTTGGACTAACGATATTGGAGTGTGCTACTGGTAAATTTCCATATAATGTCAACGAAGGCCCAGCCAATCTCATGCTACAGATACTCGATGATCCATCACCAACACCACCAGAAG
ATGCCTATACACCAGAATTTTGTTCCTTCATAAATGATTGCTTGAGGAAAGATGCTGATGCAAGGCCTACATGCGAGCAGCTTTTGTCACACGCATTTATCAAGAGGTATGAGCAA
ACTGGTGTGGACTTGGCAGCATATGTCAGGGGTGTTGTTAATCCAACAGAGAGATTAAAGCAAATAGCAGAGATGCTTGCTGTTCATTATTACCTCCTTTTTAATGGCTCTGAAGG
ACCTTGGAATTATATGAAGACATTCTACAAGGAGGAATCATCTTTCAGTTTTTCAGGGAATGTGTATGTCGGACAAAGTGCCATATTTGATACTTTATCAAATATAAGAAAGAAGT
TGAAAGGTGACCGGCCTAGAGAAAAAATTGTTCATGTTGTTGAGAAGCTACATTGCCGCGCGAATGGGGAAACAGAAATTGCTATCCGTGTGTCTGGATCATTCATCACGGGCAAC
CAATTCCTGATATTTGGTGAAGGGTTGCAAGCTGAAGGGATGCCCAGCTTGGAGGAAATTGATATTGACATTCCAAGCAAGCGGGTTGGCCAGTTCCGGGAGCAGTTTACCGTGCA
TCCAGGGACTTCCATGGGATGCTACTACATAGCAAAGCAAGATCTCTACATCGTCCAGTCATGA
```

ERA8

```
ATGTCGGGGCTAGAGGAGTTGAAGAAGAAGCTGCAGCCCTTGCTGTTCGACGACACGGACAAGGGCGGCGTCAGTACCCGGGTTCCCTTCCCGGAGGATACATGCGATTCCTATGT
GGTGTCTGATGGTGGAACGATAAATTTACTGAGTAGATCGTTTGGTGAGTATAACATCAATGAGCATGGCTTTCATAAGCGAAGCACCGGACCAGAAGAGCCAGATAGCGGTGAGA
AGGCATACCGATGTGCCTCTGAAGACATGCACATATTTGGTCCCATTGGGAATGGAGCAAGCAGTGTCGTGCAGAGAGCTATTTTCATACCAGTTCATCGAATTCTGGCCTTGAAG
AAGATAAACATATTTGAGAAGGAGAAAAGGCAACAAATTCTTAATGAGATGAGAACATTATGTGAAGCAAGTTGCTATCCTGGTTTAGTTGAATTCCAGGGTGCATTTTACATGCC
TGATTCTGGACAAATAAGCATTGCCCTTGAATACATGGATGGTGGCTCTTTAGCAGACGTTATAAAAGTCAAGAAATCAATACCAGAGCCAGTTCTTGCACATATGCTACAGAAAG
TGTTACTTGGCCTGAAGTACTTGCATGAAGTAAGACATCTAGTGCATAGAGATCTAAAGCCAGCAAATTTACTGGTAAAACTTAAGGGCGAAGCAAAAATTACAGATTTTGGTGTA
AGTGCTGGTTTGGACAATACAATGGCTATGTGTGCTACCTTTGTAGGCACTGTGACATATATGTCACCTGAGAGAATCCGCAATGAGAACTACTCCTATGCTGCTGATATTTGGAG
TCTTGGACTAACGATATTGGAGTGTGCTACTGGTAAATTTCCATATAATGTCAACGAAGGCCCAGCCAATCTCATGCTACAGATACTCGATGATCCATCACCAACACCACCAGAAG
ATGCCTATACACCAGAATTTTGTTCCTTCATAAATGATTGCTTGAGGAAAGATGCTGATGCAAGGCCTACATGCGAGCAGCTTTTGTCACACGCATTTATCAAGAGGTATGAGCAA
ACTGGTGTGGACTTGGCAGCATATGTCAGGGGTGTTGTTAATCCAACAAAGAGATTAAAGCAAATAGCAGAGATGCTTGCTGTTCATTATTACCTCCTTTTTAATGGCTCTGAAGG
ACCTTGGAATTATATGAAGACATTCTACAAGGAGGAATCATCTTTCAGTTTTTCAGGGAATGTGTATGTCGGACAAAGTGCCATATTTGATACTTTATCAAATATAAGAAAGAAGT
TGAAAGGTGACCGGCCTAGAGAAAAAATTGTTCATGTTGTTGAGAAGCTACATTGCCGCGCGAATGGGGAAACAGAAATTGCTATCCGTGTGTCTGGATCATTCATCACGGGCAAC
CAATTCCTGATATTTGGTGAAGGGTTGCAAGCTGAAGGGATGCCCAGCTTGGAGGAAATTGATATTGACATTCCAAGCAAGCGGGTTGGCCAGTTCCGGGAGCAGTTTACCGTGCA
TCCAGGGACTTCCATGGGATGCTACTACATAGCAAAGCAAGATCTCTACATCGTCCAGTCATGA
```

b ■ Catalytic domain of Plant dual-specificity Mitogen-Activated Protein Kinase Kinases ■ Nuclear transport factor 2 (NTF2) ■ EMS mutation

Zak WT

```
MSGLEELKKKLQPLLFDDTDKGGVSTRVPFPEDTCDSYVVSDGGTINLLSRSFGEYNINEHGFHKRSTGPEEPDSGEKAYRCASEDMHIFGPIGNGASSVVQRAIFIPVHRILALK
KINIFEKEKRQQILNEMRTLCEASCYPGLVEFQGAFYMPDSGQISIALEYMDGGSLADVIKVKKSIPEPVLAHMLQKVLLGLKYLHEVRHLVHRDLKPANLLVKLKGEAKITDFGV
SAGLDNTMAMCATFVGTVTYMSPERIRNENYSYAADIWSLGLTILECATGKFPYNVNEGPANLMLQILDDPSPTPPEDAYTPEFCSFINDCLRKDADARPTCEQLLSHAFIKRYEQ
TGVDLAAYVRGVVNPTERLKQIAEMLAVHYYLLFNGSEGPWNYMKTFYKEESSFSFSGNVYVGQSAIFDTLSNIRKKLKGDRPREKIVHVVEKLHCRANGETEIAIRVSGSFITGN
QFLIFGEGLQAEGMPSLEEIDIDIPSKRVGQFREQFTVHPGTSMGCYYIAKQDLYIVQS-
```

ERA8

```
MSGLEELKKKLQPLLFDDTDKGGVSTRVPFPEDTCDSYVVSDGGTINLLSRSFGEYNINEHGFHKRSTGPEEPDSGEKAYRCASEDMHIFGPIGNGASSVVQRAIFIPVHRILALK
KINIFEKEKRQQILNEMRTLCEASCYPGLVEFQGAFYMPDSGQISIALEYMDGGSLADVIKVKKSIPEPVLAHMLQKVLLGLKYLHEVRHLVHRDLKPANLLVKLKGEAKITDFGV
SAGLDNTMAMCATFVGTVTYMSPERIRNENYSYAADIWSLGLTILECATGKFPYNVNEGPANLMLQILDDPSPTPPEDAYTPEFCSFINDCLRKDADARPTCEQLLSHAFIKRYEQ
TGVDLAAYVRGVVNPTKRLKQIAEMLAVHYYLLFNGSEGPWNYMKTFYKEESSFSFSGNVYVGQSAIFDTLSNIRKKLKGDRPREKIVHVVEKLHCRANGETEIAIRVSGSFITGN
QFLIFGEGLQAEGMPSLEEIDIDIPSKRVGQFREQFTVHPGTSMGCYYIAKQDLYIVQS-
```

Fig. S6 –WT and *ERA8* coding sequence of *TaMKK3-A*. **a**) Coding region nucleotide sequences include the *TaMKK3-A* C660A mutation (blue) found in Torada et al. (2016) and the *TaMKK3-A* G1093A EMS mutation (purple) found between Zak WT and *ERA8*. **b**) The protein sequence of *TaMKK3-A* contains a Catalytic domain of Plant dual-specificity Mitogen-Activated Protein Kinase Kinases (light blue) and a Nuclear transport factor 2 (NTF2; green). Note this is reverse complement relative to the reference scaffold.
