# Supplementary material for: Exome sequencing of bulked segregants identified a novel TaMKK3-A allele linked to the wheat ERA8 ABA-hypersensitive germination phenotype
Source: Theor Appl Genet. 2020 Jan 28;133(3):719–36. doi: 10.1007/s00122-019-03503-0 (PMC7021667; doi:10.1007/s00122-019-03503-0)
Supplement: Supplementary file 2 — Table S1—Summary of Zak/ZakERA8 ABA phenotyping conditions. Table S2—Chi-squared analysis of Zak/ZakERA8 BC3F2:3 X5.2. Table S3—Exome capture sequencing quality and statistics. Table S4—Primers created from the GBS and exome capture identified SNPs. Table S5—Chi-squared analysis of Zak/ZakERA8 BC3F2:3 X5.1 and X5.2. Table S6—Louise/ZakERA8 linkage group summary. Table S7—Genes in the ERA8 4A region. Table S8—Summary of Louise/ZakERA8 ABA phenotyping conditions. Table S9—Significant QTL of ABA sensitivity, height, and heading date for Louise/ZakERA8. Table S10—Genes differentially expressed between Zak and ERA8 (PDF 425 kb) [file 122_2019_3503_MOESM2_ESM.pdf]

SUPPLEMENTAL MATERIAL 2: TABLE S1 – S10

Martinez SA, Shorinola O, Conselman S, See D, Skinner DZ, Uauy C, and Steber CM (2020) Exome sequencing of bulked segregants identified a novel *TaMKK3-A* allele linked to the wheat *ERA8* ABA-hypersensitive germination phenotype. Theor Appl Genet

Correspondence: Camille M. Steber
email: camille.steber@usda.gov
phone: (509) 335-2887

Table S1 - Summary of Zak/Zak*ERA8* ABA phenotyping conditions
Table S2 - Chi-squared analysis of Zak/Zak*ERA8* BC3F2:3 X5.2
Table S3 - Exome capture sequencing quality and statistics
Table S4 - Primers created from the GBS and exome capture identified SNPs
Table S5 - Chi-squared analysis of Zak/Zak*ERA8* BC3F2:3 X5.1 and X5.2
Table S6 - Louise/Zak*ERA8* linkage group summary
Table S7 - Genes in the *ERA8* 4A region
Table S8 - Summary of Louise/Zak*ERA8* ABA phenotyping conditions
Table S9 - Significant QTL of ABA sensitivity, height, and heading date for Louise/Zak*ERA8*
Table S10 - Genes differentially expressed between Zak and *ERA8*

Table S1 ABA sensitivity for the Zak/Zak*ERA8* $BC_3F_{2:3}$ population across different seed lots

**Location**	**Year**	**n** [a]	**b. rep** [b]	**ABA (μM)**	**AR** [c] **(days)**	**Imb.** [d] **(days)**	**Mean ± SD** [d]		**Storage in -20 °C (months)**
							**PG** [d]	**GI** [d]	
5.2 BSA	2015	15	3	2	35	5	12.6 ± 8.5	-	4
		17	3	2	35	5	79.9 ± 13	-	4
		ZakERA8	16	2	35	5	8.3 ± 7.2	-	4
		Zak	16	2	35	5	90.8 ± 15	-	4
5.2a	2015	242	2	2	35	5	42.6 ± 21	0.21 ± 0.1	17
		ZakERA8	8	2	35	5	8.3 ± 4.3	0.03 ± 0.0	17
		Zak	8	2	35	5	90.8 ± 12	0.55 ± 0.0	17
5.1	2017	122	1	10	42, 59	4	43.2 ± 25	0.29 ± 0.1	6
		ZakERA8	5	10	42, 59	4	54.3 ± 15	0.14 ± 0.1	6
		Zak	5	10	42, 59	4	89.3 ± 5.5	0.40 ± 0.1	6
5.2b	2017	60	3	5	41	4	68.5 ± 17	0.42 ± 0.1	3
		ZakERA8	12	5	41	4	12.7 ± 5.2	0.08 ± 0.0	3
		Zak	12	5	41	4	90.5 ± 3.5	0.62 ± 0.0	3

[a] number (n) of backcross lines tested

[b] Biological replicates (b. rep) per backcross or parental line.

[c] Days after-ripened (AR)

[d] The number of days imbibed (Imb.) that was used for bulk segregant analysis (BSA) or fine mapping

[e] Raw mean and standard deviation (SD) of percent germination (PG) and germination index (GI) across the backcross population, Zak, or *ERA8*.

Table S2 Segregation analysis of BC_3F_2 seed germination on 2uM ABA

**Genotype**	**n**[a]	**Gen.**[b]	**Germ.**[c]	**Not Germ.**[c]	**χ²**			**p-value**		
					**3:1**	**1:3**	**1:2:1**	**3:1**	**1:3**	**1:2:1**
5.2a segregating	300	BC_3F_2	154	146	140.7	36.6	1.62	<0.001	<0.001	**0.20**
+/+	60	parent[e]	54	6						
ERA8/*ERA8*	60	parent[e]	0	60						
+/*ERA8*	8	BC_2F_1	4	4						
F_2 Expected[d]	300				68	203	143			

[a] Number of seeds tested for germination, after-ripened for 5 weeks past physiological maturity; df = 1

[b] Generation of seeds tested

[c] Number of seeds that had germinated (Germ.) and not germinated (Not Germ.) after 5 days of imbibition on 2μM (+/-) ABA.

[d] Number of seeds expected to germinate after 5 days of imbibition for each single gene segregation ratio.

[e] Zak (+/+) and *ERA8* (*ERA8*/*ERA8*) parental lines were used to generate cross 5; Parents were grown at the same time as the F_1 plants.

Table S3 Sequencing read count and quality statistics from exome capture

**Sample Name**	**Number of Reads**	**Mean Q30 to base Read 1**	**Mean Q30 to base Read 2**
ERA8 Parent	60,514,766	125	125
*ERA8*_Bulk	57,332,604	126	125
WT Parent	48,792,615	126	125
WT_Bulk	67,097,197	126	125

Table S4 *ERA8* primers created from the GBS and Exome capture identified SNPs

Name	Allele	Sequence	Tag	Source
SNP_1	WT	GAAGGTGACCAAGTTCATGCTaCtctttctcatgagctctttgaG	FAM	Exome
	ERA8	GAAGGTCGGAGTCAACGGATTaCtctttctcatgagctctttgaA	HEX	
	C	tcgcgttcatcatctacctG	-	
SNP_2	WT	GAAGGTGACCAAGTTCATGCTctgaaatgCctgcaagatgaG	FAM	Exome
	ERA8	GAAGGTCGGAGTCAACGGATTctgaaatgCctgcaagatgaA	HEX	
	C	ggaaggccaaaatagcgacttA	-	
SNP_4	WT	GAAGGTGACCAAGTTCATGCTaaactcgacGaaaTtgcCaTC	FAM	Exome
	ERA8	GAAGGTCGGAGTCAACGGATTaaactcgacGaaaTtgcCaTT	HEX	
	C	aatcaacaCcattttcatttcacaG	-	
SNP_5	WT	GAAGGTGACCAAGTTCATGCTgtggagtgTctgcaagatcG	FAM	Exome
	ERA8	GAAGGTCGGAGTCAACGGATTgtggagtgTctgcaagatcA	HEX	
	C	tatgcactcaccacgccA	-	
SNP_6	WT	GAAGGTGACCAAGTTCATGCTCacaaactgtgctatcccgG	FAM	Exome
	ERA8	GAAGGTCGGAGTCAACGGATTCacaaactgtgctatcccgA	HEX	
	C	tggtagcagttgtcttgacC	-	
SNP_7	WT	GAAGGTGACCAAGTTCATGCTggcttgcgggtaaaagagG	FAM	Exome
	ERA8	GAAGGTCGGAGTCAACGGATTggcttgcgggtaaaagagA	HEX	
	C	tctttgttttataattgcagtgcaC	-	
SNP_8	WT	GAAGGTGACCAAGTTCATGCTtgTtAGatgtGaccttacctctcaG	FAM	Exome
	ERA8	GAAGGTCGGAGTCAACGGATTtgTtAGatgtGaccttacctctcaA	HEX	
	C	cgagatgagcaaggaggtgg	-	
SNP_9	WT	GAAGGTGACCAAGTTCATGCTCggttctgcgttaccccatG	FAM	Exome
	ERA8	GAAGGTCGGAGTCAACGGATTCggttctgcgttaccccatA	HEX	
	C	agcaaatgCcgCggatcG	-	
SNP_10	WT	GAAGGTGACCAAGTTCATGCTCggatcacgaTcgccttctcC	FAM	Exome
	ERA8	GAAGGTCGGAGTCAACGGATTCggatcacgaTcgccttctcT	HEX	
	C	TgTgctcctcGtcCtGAC	-	
SNP_17	WT	GAAGGTGACCAAGTTCATGCTCCTCTGCTATTTGCTTTAATCTCTc	FAM	Exome
	ERA8	GAAGGTCGGAGTCAACGGATTCCTCTGCTATTTGCTTTAATCTCTt	VIC	
	C	GGACTTGGCAGCATATGTCA	-	
SNP_20	WT	GAAGGTGACCAAGTTCATGCTCtcctgctcctgcttccgG	FAM	Exome
	ERA8	GAAGGTCGGAGTCAACGGATTCtcctgctcctgcttccgA	HEX	
	C	cggcctcacttgcagaaaac	-	
SNP_29	WT	GAAGGTGACCAAGTTCATGCTgtgtacgcGcgCtactgC	FAM	Exome
	ERA8	GAAGGTCGGAGTCAACGGATTgtgtacgcGcgCtactgT	HEX	
	C	ccatgatctccagcgacagA	-	
SNP_30	WT	GAAGGTGACCAAGTTCATGCTggtataaacatcagaatccctgtcC	FAM	Exome
	ERA8	GAAGGTCGGAGTCAACGGATTggtataaacatcagaatccctgtcT	HEX	
	C	ccggcatcttcTGtattaacatacA	-	
PHS1[a]	Res	GAAGGTGACCAAGTTCATGCTTTTTGCTTCGCCCTTAAGG	FAM	-
	Susc	GAAGGTCGGAGTCAACGGATTTTTTTGCTTCGCCCTTAAGT	HEX	
	C	GCATAGAGATCTAAAGCCAGCA	-	
A7575	WT	GAAGGTCGGAGTCAACGGATTCaAActacacactcgtcgggA	HEX	GBS
	ERA8	GAAGGTGACCAAGTTCATGCTCaAActacacactcgtcgggG	FAM	
	C	ccctgcagcagaggacatC	-	
A7946	*ERA8*	GAAGGTCGGAGTCAACGGATTCaAactacacactcgtcgggA	HEX	GBS
	Ref	GAAGGTGACCAAGTTCATGCTCaAactacacactcgtcgggG	FAM	
	C	ccctgcagcagaggacatC	-	

[a] PHS1 KASP primers were developed by Shorinola et al. (2017)

[b] KASP tags (FAM and HEX) are already included in the primer sequence and indicated by grey text

Table S5 Segregation analysis of Zak/*ZakERA8* BC_3F_2 X5.1, X5.2, and X5.3 germination on ABA

**Genotype**	**n**[a]	**Gen.**[b]	**Germ.**[c]	**Not Germ.**[c]	**χ^2**			**p-value**		
				**F_2**	**3:1**	**1:3**	**1:2:1**	**3:1**	**1:3**	**1:2:1**
				**F_3**	**0.625:0.375**	**0.375:0.625**	**0.375:0.25:0.375**	**0.625:0.375**	**0.375:0.625**	**0.375:0.25:0.375**
56d AR | 10uM ABA | 4d imb.										
**X5.1**	1311	BC_3F_3	588	723	18.6	39.8	0.08	<0.001	<0.001	0.78
WT/WT	54	parent [e]	39	15						
ERA8/ERA8	60	parent [e]	9	51						
WT/*ERA8*	8	BC_2F_1	4	4						
F_3 Expected [d]	1311				666	478	593			
35d AR | 2uM ABA | 4d imb.										
**X5.2a**	7361	BC_3F_3	3974	3387	107.0	677.3	55.3	<0.001	<0.001	<0.001
WT/WT	120	parent [e]	109	11						
ERA8/ERA8	120	parent [e]	10	110						
WT/*ERA8*	8	BC_2F_1	4	4						
F_3 Expected [d]	7361				4409	2885	3655			
49d AR | 5uM ABA | 5d imb.										
**X5.2b**	583	BC_3F_3	340	243	30.8	23.5	0.84	<0.001	<0.001	0.36
WT/WT	59	parent [e]	59	0						
ERA8/ERA8	52	parent [e]	9	43						
WT/*ERA8*	8	BC_2F_1	4	4						
F_3 Expected [d]	583				402	282	329			
56d AR | 10uM ABA | 4d imb.										
**X5.3** [f]	1453	BC_3F_3	344	1109	427.5	102.8	272.2	<0.001	<0.001	<0.001
WT/WT	54	parent [e]	39	15						
ERA8/ERA8	60	parent [e]	9	51						
WT/*ERA8*	8	BC_2F_1	4	4						
F_3 Expected [d]	1453				738	530	657			

[a] Number of seeds tested for germination, after-ripened for 5 weeks past physiological maturity; df = 1

[b] Generation of seeds tested

[c] Number of seeds that had germinated (Germ.) and not germinated (Not Germ.) after 5 days of imbibition on 2μM (+/-) ABA.

[d] Number of seeds expected to **germinate** after 5 days of imbibition for each single gene segregation ratio.

[e] Zak WT (+/+) and Zak *ERA8* (-/-) parental lines used to generate cross 5; Grown at the same time as the F_1 plants.

[d] Note that X5.3 was not used in any analyses since it appears to not have a 1:2:1 segregation.

Table S6 Summary of the Louise/Zak*ERA8* RIL population GBS linkage groups

Chrm	No. Groups [a]	Total Number of Markers	Map Distance (cM)
1A	1	231	534
1B	3	147	334, 41, 88
1D	2	57	73, 193
2A	2	98	46, 272
2B	1	49	270
2D	1	57	483
3A	4	180	296, 89, 33, 118
3B	1	64	241
3D	2	46	97, 153
4A	3	224	279, 134, 30
4B	2	130	294, 55
4D	1	11	122
5A	2	62	543, 42
5B	3	84	138, 117, 38
5D	2	27	239, 113
6A	2	169	323, 168
6B	2	39	167, 72
6D	3	34	62, 167, 23
7A	2	238	458, 272
7B	2	206	227, 40
7D	3	61	268, 78, 116
unknown	1	20	105
total	45	2,234	-

[a] Linkage groups were assigned to chromosomes based on the majority of markers aligned to one chromosome on the RefSeqv1.0 wheat genome (IWGSC 2018).

Table S7 Genes in the *ERA8* interval on chromosome 4A between SNP_20 and SNP_29 markers

Gene Model Name	Annotation	Start Position	Orientation	EMS SNP	Primer
TraesCS4A01G299700	High affinity cationic amino acid transporter 1, Uncharacterized protein	597,908,536	+	C → T	SNP_6
TraesCS4A01G311100	Poly(A) RNA polymerase GLD2-A, Uncharacterized protein	603,446,405		C → T	SNP_19
TraesCS4A01G311800	Protein UPSTREAM OF FLC	603,496,846	-	-	
TraesCS4A01G311900	Antimicrobial peptide MBP-1 related (LEM1)	603,504,474	+	-	
TraesCS4A01G312000	TRAF-like superfamily protein	603,507,001	+	-	
TraesCS4A01G312100	Hydroxyproline-rich glycoprotein family protein	603,510,936	-	-	
TraesCS4A01G312200	GSK1 transcription factor 1	603,530,872	-		
		603,532,130		C → T	SNP_20
TraesCS4A01G312300	Calcium-dependent lipid-binding domain protein	603,533,711	-	-	
TraesCS4A01G312400	AT hook motif DNA-binding family protein	603,539,435	-	-	
TraesCS4A01G312500	LOB domain-containing protein, putative	603,634,586	-	-	
TraesCS4A01G312600	Peptidyl-prolyl cis-trans isomerase	603,637,101	+	-	
TraesCS4A01G312700	Aquaporin-like protein	603,666,065	+	-	
TraesCS4A01G312800	Disease resistance protein (NBS-LRR class) family	603,674,385	+	-	
TraesCS4A01G312900	Disease resistance protein (NBS-LRR class) family	603,698,121	+	-	
TraesCS4A01G313000	Retrovirus-related Pol polyprotein from transposon TNT 1-94	603,699,642	-	-	
TraesCS4A01G313100	Leucine rich repeat	603,726,600	-	-	
TraesCS4A01G313200	YUCCA9	603,980,902	-	-	
TraesCS4A01G313300	PM19-A1	604,055,487	-	-	
TraesCS4A01G313400	PM19-A2	604,068,519	+	-	
TraesCS4A01G313500	Myosin-J-Protein	604,076,529	+	-	
TraesCS4A01G313600	Ubiquintin Congujating Enzyme E2-23	604,101,314	+	-	
TraesCS4A01G313700	ACC Oxidase -1 Like	604,104,116	-	-	
TraesCS4A01G313800	B3-domain-containing protein	604,107,899	+	-	
TraesCS4A01G313900	LRR receptor-Like Kinase Serine/threonine-protein Kinase	604,209,020	-	-	
-	pseudogene	604,315,235		C → T	SNP_33
TraesCS4A01G314000	ACC Oxidase -1 Like	604,594,773	-	-	
TraesCS4A01G314100	Anthocyanin 5-aromatic acyltransferase	604,659,700	+	-	
TraesCS4A01G314200	LRR receptor-like kinase	604,672,359	+	-	
TraesCS4A01G314300	Disease resistance protein RPM1	604,772,917	+	-	
TraesCS4A01G314400	Disease resistance protein RPM1	604,849,387	-	-	
TraesCS4A01G314500	ERF-1B-Like	604,856,919	+	-	
TraesCS4A01G314600	ERF-1B-Like	604,940,912	+	-	
TraesCS4A01G314700	BTB/POZ/MATH-domain protein	605,018,735	-	-	
TraesCSU01G167000 [a]	TaMKK3-A	-		G → A	SNP_17
TraesCS4A01G314800	transcription factor, putative (Protein of unknown function, DUF547)	605,024,348	+	-	
TraesCS4A01G314900	Sugar transporter, putative	605,087,172	-	-	
TraesCS4A01G315000	Serine/threonine-protein phosphatase	605,137,125	-	-	
-	low confidence gene	605,375,951		G → A	SNP_34
TraesCS4A01G315100	Protein kinase	605,559,326	-	-	
TraesCS4A01G315200	OTU domain-containing protein	605,640,606	+	-	

TraesCS4A01G315300	Trihelix transcription factor	605,644,924	+	-	
TraesCS4A01G315400	Early nodulin-like protein	605,650,522	-	-	
TraesCS4A01G315500	60 kDa chaperonin	605,656,215	+	-	
TraesCS4A01G315600	Mediator of RNA polymerase II transcription subunit 15a	605,663,094	-	-	
TraesCS4A01G315700	RNA polymerase sigma factor	605,711,154	+	-	
TraesCS4A01G315800	Mediator of RNA polymerase II transcription subunit 13	605,717,570	+	-	
TraesCS4A01G315900	ABC transporter G family member	605,744,452	+	-	
TraesCS4A01G316000	Cytoplasmic polyadenylation element-binding protein 4	606,116,294	+	-	
TraesCS4A01G316100	F-box family protein	606,335,446	+	-	
TraesCS4A01G316200	Heavy metal-associated protein	606,345,914	-	-	
TraesCS4A01G316300	La-related protein	606,361,307	+	-	
TraesCS4A01G316400	GDSL esterase/lipase	606,370,282	+	-	
TraesCS4A01G316500	GDSL esterase/lipase	606,397,403	+	-	
TraesCS4A01G316600	Protein DETOXIFICATION	606,411,251	+	-	
TraesCS4A01G316700	Avr9/Cf-9 rapidly elicited protein	606,523,442	+	-	
TraesCS4A01G316800	WD40 repeat-containing protein	606,536,121	-	-	
TraesCS4A01G316900	phosphotransferases/inositol or phosphatidylinositol kinase	606,585,463	+	-	
TraesCS4A01G317000	Bifunctional inhibitor/lipid-transfer protein/seed storage 2S albumin-like protein	606,591,102	+	-	
TraesCS4A01G317100	DNA translocase FtsK	606,593,057	-	-	
TraesCS4A01G317200	Harpin-induced protein 1 (Hin1), putative	606,608,730	+	-	
TraesCS4A01G317300	Galactose-6-phosphate isomerase subunit LacB	606,643,500	-	-	
TraesCS4A01G317400	Mannonate dehydratase	606,647,800	-	-	
TraesCS4A01G317500	Kinase family protein	606,761,092	+	-	
TraesCS4A01G317600	Kinase family protein	606,796,989	+	-	
TraesCS4A01G317700	Oxidoreductase/transition metal ion-binding protein	606,811,790	+	-	
TraesCS4A01G317800	Exportin-1	607,045,327	+	-	
TraesCS4A01G317900	Cytokinin riboside 5'-monophosphate phosphoribohydrolase	607,177,351	-	-	
TraesCS4A01G318000	Plant invertase/pectin methylesterase inhibitor superfamily	607,178,696	+	-	
TraesCS4A01G318100	Cytokinin riboside 5'-monophosphate phosphoribohydrolase	607,261,062	-	-	
TraesCS4A01G318200	F-box only 46	607,270,018	+	-	
TraesCS4A01G318300	Leucine-rich repeat receptor-like protein kinase family protein	607,270,941	-	-	
TraesCS4A01G318400	ATP-dependent zinc metalloprotease FtsH	607,309,139	+	-	
TraesCS4A01G318500	DNA topoisomerase	607,374,419	-	-	
TraesCS4A01G318600	EMBRYO SURROUNDING FACTOR 1-like protein 8	607,378,421	+	-	
TraesCS4A01G318700	Pentatricopeptide repeat-containing protein At1g19720	607,417,280	+	-	
TraesCS4A01G318800	carbohydrate esterase, putative (DUF303)	607,427,390	-	-	
TraesCS4A01G318900	NAD/NADP-dependent betaine aldehyde dehydrogenase	607,432,144	+	-	
TraesCS4A01G319000	PGR5-like protein 1A, chloroplastic	607,633229	+	-	
-	-	607,886,990		-	Barc170
TraesCS4A01G319100	Gibberellin 20 oxidase	608,043,459		G → A	SNP_29
TraesCS4A01G325400	Pentatricopeptide repeat-containing protein; Pyridine nucleotide-disulphide oxidorectuse	613,268,437		G → A	SNP_30

[a] The current reference genome (RefSeq v1.0; IWGSC 2018) aligned *TaMKK3-A* to the unknown chromosome. The estimated location of *TaMKK3-A* on chromosome 4A is based on Shorinola et al. (2017).

Table S8 ABA sensitivity for the Louise/Zak*ERA8* RIL population was tested across three environments

Location	Year	n[a]	t rep[b]	ABA (μM)	AR[c] (days)	Mean ± SD[d]					
						Day 1	Day 2	Day 3	Day 4	Day 5	GI
Greenhouse	2013	225	3	5	49	48.9 ± 29	71.9 ± 25	76.0 ± 22	78.8 ± 21	81.2 ± 19	0.71 ± 0.2
(E1)		*ERA8*	9	5	49	26.7 ± 21	68.9 ± 21	74.4 ± 18	78.1 ± 16	81.1 ± 16	0.66 ± 0.2
		Louise	9	5	49	70.4 ± 12	81.9 ± 13	86.3 ± 12	88.9 ± 10	91.9 ± 8	0.84 ± 0.1
Field	2014	181	3	2	42	22.3 ± 19	52.5 ± 25	63.3 ± 25	69.8 ± 25	74.9 ± 25	0.56 ± 0.2
(E2)		*ERA8*	3	2	42	0.0 ± 0	5.6 ± 5	13.3 ± 12	17.8 ± 13	20.0 ± 12	0.11 ± 0.1
		Louise	3	2	42	24.3 ± 8	44.4 ± 13	69.8 ± 18	77.8 ± 22	87.0 ± 11	0.58 ± 0.2
Field	2015	190	3	2	48	23.1 ± 22	71.5 ± 27	78.2 ± 24	82.2 ± 23	84.8 ± 21	0.68 ± 0.2
(E3)		*ERA8*	3	2	48	5.6 ± 2	37.8 ± 23	43.3 ± 30	50.0 ± 27	53.3 ± 30	0.38 ± 0.2
		Louise	3	2	48	27.8 ± 4	81.1 ± 10	86.7 ± 6	87.8 ± 7	88.9 ± 5	0.75 ± 0.1

[a] number (n) of recombinant inbred lines (RIL) tested

[b] technical replicates per RIL or parental line

[c] Days after-ripened (AR)

[d] Raw mean and standard deviation (SD) of percent germination (PG) and germination index (GI) across the backcross population, Zak, or *ERA8*.

Table S9 Significant QTL in the Louise/Zak*ERA8* RIL population conducted with both the GBS and EMS-induce SNP markers

QTL Name	Marker	Chrm	Pos (cM)	Start [a]	End [a]	SNP Position	LOD	Trait [b]	Favorable Allele [c]
QABA.wsu-4A.1	A11335	4A	271.8	83,469,184	83,469,281	85	3.97	E3 D1	C/**T**
-	A14187	4A	321.6	195,745,016	195,745,115	31	4.01	E2 D2	C/**T**
-	A13781	4A	320.8	195,745,016	195,745,085	31	3.66	E2 D2	C/**T**
QABA.wsu-4A.5	A12726	4A	295.9	216,478,520	216,478,451	47	4.8	E2 D1	G/**A**
	A10010	4A	301	316,676,795	316,676,894	34	5.16	E2 D1	G/**A**
	A9539	4A	301.8	316,676,795	316,676,864	34	5.02	E2 D1	G/**A**
	A25632	4A	298.3	509,248,865	509,248,964	88	5.12	E2 D1	A/**G**
	A14030	4A	299.7	526,310,792	526,310,693	49	4.19	E2 D1	C/**T**
	A25258	4A	299.1	534,315,662	534,315,563	16	4.73	E2 D1	T/**C**
	A15895	4A	297.3	534,325,792	534,325,693	47, 87	4.98	E2 D1	G, C / **C, T**
QABA.wsu-4A.2	SNP_5	4A	325.5	-	-	-	4.45	E2 D2	G/**A**
	SNP_9	4A	326	533,446,137	-	-	4.23	E2 D2	G/**A**
	SNP_4	4A	326.2	532,074,134	-	-	4.32	E2 D2	C/**T**
	SNP_10	4A	328.4	586,452,395	-	-	4.41	E2 D2	C/**T**
-	A18189	4A	323.3	566,902,964	566,903,063	95	3.68	E2 D2	A/**G**
QABA.wsu-4A.6	A16514	4A	294.7	573,801,680	573,801,581	52	4.31	E2 D1	C/**T**
	A16172	4A	294	573,801,680	573,801,611	52	4.31	E2 D1	C/**T**
	A2917	4A	292.5	577,057,065	577,056,996	49	4.67	E2 D1	G/**C**
QABA.wsu-4A.8	A29481	4A	342.8	595,374,988	595,374,889	28	4.17	E3 GI, D3	T/**C**
	A29272	4A	342	595,374,988	595,374,919	28	3.49	E3 GI	T/**C**
	A22222	4A	338.2	602,250,029	602,249,930	64	3.69	E3 GI	G/**A**
QABA.wsu-4A.4	SNP_20	4A	353.5	603,532,130	-	-	4.22	E1 D1	**G**/A
	TaMKK3-A	4A	356.2	-	-	-	5.35	E1 D1	**C**/A
	SNP_17	4A	356.2	-	-	-	5.35	E1 D1	**G**/A
	SNP_29	4A	358.7	608,044,262	-	-	4.44	E1 D1	**C**/T
QABA.wsu-4A.10	A190	4A	439.1	623,366,977	623,366,908	17	4.07	E3 D4-5, GI	**G**/T
QABA.wsu-4A.7	A28342	4A	422.9	645,311,558	645,311,459	91	4.46	E2 D2-5, GI	**A**/C
QABA.wsu-4A.9	A23553	4A	141.1	658,854,423	658,854,492	55	3.96	E2 D1	**G**/A
Qhd.wsu-4A	A23913	4A	50.93	658,854,423	658,854,522	55	4.87	Heading	G/A
Qhei.wsu-7B	A2003	7B	252.3	7,574,338	7,574,239	84	4.49	Height	T/A

[a] The GBS markers was aligned to the RefSeqv1.0 reference genome (IWGSC 2018) and the start and end nucleotide position is reported with the SNP position within the sequence.

[b] Significant QTL for the following traits are indicated by environment (E1, E2, or E3) followed by the percent germination after n days (D1-D5) of imbibition or germination index (GI). Significant QTL for heading date and height are also reported.

[c] Favorable alleles (underlined) decrease germination percent or index. Louise contributed the **first** and *ERA8* the **second** allele.

Table S10 Unique genes differentially expressed between WT and *ERA8.* Highly differentially expressed genes are listed from the whole genome that were a) *ERA8* upregulate / WT downregulated (+ beta value) or b) *ERA8* downregulated / WT upregulated (- beta value). c) Differential expression of all unique genes located on chromosome 4A are listed with the EMS-induced SNP markers indicated in grey rows for reference.

	Gene ID [a]	Annotation	Chrm	Start	End	beta [b]
**a)**	TraesCS2B01G524400	-	2B	718,960,478	718,968,471	**7.08**
	TraesCS3B01G322600	-	3B	521,789,364	521,797,431	**5.69**
	TraesCS2D01G000600	Polycomb group protein VERNALIZATION 2	2D	278,541	286,171	**5.26**
	TraesCS6A01G338800	DNA (Cytosine-5-)-methyltransferase	6A	572,133,054	572,138,876	**5.20**
	TraesCS7D01G269200	Reticulocyte-binding protein 2 a	7D	253,341,441	253,348,906	**5.05**
**b)**	TraesCS7B01G229200	-	7B	430,829,130	430,842,036	**-5.20**
	TraesCS2D01G497100	Protein UXT-like protein	2D	593,215,038	593,217,521	**-5.24**
	TraesCS1A01G374800	Zinc finger protein-like	1A	549,837,796	549,847,148	**-5.39**
	TraesCS3B01G338100	-	3B	544,780,837	544,793,535	**-5.42**
	TraesCS7B01G484300	DUF789 family protein	7B	741,573,444	741,579,514	**-5.57**
	TraesCS2D01G500500	-	2D	595,157,820	595,161,251	**-5.58**
**c)**	TraesCS4A01G016000	Transcription factor	4A	10,164,187	10,165,250	-1.09
	TraesCS4A01G021100	F-box and associated interaction domains-containing protein	4A	14,340,239	14,344,018	1.47
	TraesCS4A01G021900LC	Protein yippee-like	4A	17,974,706	17,977,539	-0.87
	TraesCS4A01G022000LC	Retrotransposon protein, putative, unclassified	4A	17,978,744	17,981,991	**-1.80**
	TraesCS4A01G029300LC	Protein FAR1-RELATED SEQUENCE 5	4A	25,211,087	25,215,287	**4.17**
	TraesCS4A01G041700	-	4A	35,181,518	35,183,648	**4.29**
	TraesCS4A01G050500	SNP_1	4A	41,115,524	-	-
	TraesCS4A01G052700LC	Sodium/hydrogen exchanger	4A	46,171,955	46,199,813	0.94
	TraesCS4A01G052800LC	Sentrin-specific protease 1	4A	46,173,178	46,174,094	**2.92**
	TraesCS4A01G079100	-	4A	80,840,552	80,844,726	**4.10**
	TraesCS4A01G087400	SNP_2	4A	91,795,916	-	-
	TraesCS4A01G092100	Heat-shock protein, putative	4A	98,785,386	98,787,839	0.80
	TraesCS4A01G097400	Tryptophan synthase alpha chain	4A	108,369,445	108,371,437	-1.43
	TraesCS4A01G099900	Histone H2B	4A	112,767,092	112,767,812	-0.51
	TraesCS4A01G103900	SNP_13	4A	117,337,422	-	-
	TraesCS4A01G121600	SNP_14	4A	150,302,500	-	-
	TraesCS4A01G126600	8-amino-7-oxononanoate synthase	4A	163,345,723	163,347,867	-1.01
	TraesCS4A01G130600	NAC domain protein,	4A	173,630,224	173,632,115	-0.69
	TraesCS4A01G131600	SNP_7	4A	175,858,520	-	-
	TraesCS4A01G131700	SNP_3	4A	176,543,353	-	-
	TraesCS4A01G167000LC	Mitochondrial transcription termination factor family protein	4A	207,976,194	207,976,616	1.32
	TraesCS4A01G179300LC	2'-phosphotransferase	4A	230,997,232	231,003,186	-0.67
	TraesCS4A01G172100	SNP_8	4A	436,828,695	-	-
	TraesCS4A01G248100LC	S-adenosyl-L-methionine-dependent methyltransferases superfamily protein	4A	340,489,977	340,490,856	**2.02**
	TraesCS4A01G160600	Phosphatidate phosphatase, Lipin	4A	345,835,861	345,844,921	-1.17
	TraesCS4A01G316400LC	Serine/threonine-protein kinase	4A	465,545,872	465,547,035	0.81
	TraesCS4A01G323300LC	Cysteine desulfurase, putative, expressed	4A	474,726,464	474,739,302	-0.47
	TraesCS4A01G194800	Non-specific serine/threonine protein kinase	4A	476,977,837	476,980,583	-0.69
	TraesCS4A01G202600	Carboxypeptidase	4A	492,530,884	492,534,660	-0.70

TraesCS4A01G340900LC	MLO-like protein	4A	496,577,022	496,578,105	**2.55**
TraesCS4A01G220100	DNA polymerase	4A	522,907,385	522,921,948	-0.92
TraesCS4A01G221500	Sphingoid base hydroxylase 2	4A	527,112,529	527,113,641	**2.65**
TraesCS4A01G224300	SNP_4	4A	532,074,134	-	-
TraesCS4A01G225500	SNP_9	4A	533,446,137	-	-
TraesCS4A01G232700, or TraesCS4A01G232800	SNP_15	4A	542,033,771	-	-
TraesCS4A01G243200	RING/U-box superfamily protein	4A	553,310,509	553,314,175	0.79
TraesCS4A01G252800	Cathepsin B-like cysteine protease	4A	565,040,316	565,043,816	-1.32
TraesCS4A01G411100LC	Transposon protein, putative, mutator sub-class	4A	566,101,663	566,104,738	**3.76**
TraesCS4A01G258100	Lariat debranching enzyme	4A	570,981,287	570,985,759	0.39
TraesCS4A01G259300	Anthocyanin 5-aromatic acyltransferase	4A	572,302,274	572,303,653	0.65
TraesCS4A01G264600	UDP-glucose 6-dehydrogenase	4A	576,930,165	576,932,752	-0.60
TraesCS4A01G278800	SNP_10	4A	586,452,395	-	-
TraesCS4A01G444900LC	Peptide transporter	4A	591,496,571	591,499,205	-0.97
TraesCS4A01G288000	Argonaute protein	4A	593,194,838	593,200,481	0.99
TraesCS4A01G290300	Ankyrin repeat family protein	4A	594,180,282	594,186,698	1.12
TraesCS4A01G299700	SNP_6	4A	597,908,536	-	-
TraesCS4A01G311100	SNP_19	4A	603,446,405	-	-
TraesCS4A01G312200	SNP_20 GSK1 transcription factor 1	4A	603,532,130	-	-
TraesCSU01G167000	SNP_17 MKK3	unk	-	-	-
-	barc170	4A	607,886,990	-	-
TraesCS4A01G319100	SNP_29 GA 20-ox	4A	608,044,262	-	-
TraesCS4A01G485400LC	BED zinc finger,hAT family dimerization domain	4A	612,510,456	612,511,014	-1.26
TraesCS4A01G485500LC	BED zinc finger,hAT family dimerization domain	4A	612,511,090	612,513,756	**-2.60**
TraesCS4A01G325400	SNP_30	4A	613,286,437	-	-
TraesCS4A01G331900	Cinnamoyl-CoA reductase 4	4A	616,309,725	616,313,994	0.44
TraesCS4A01G345800	Threonine synthase 1, chloroplastic	4A	625,029,910	625,030,335	0.86
TraesCS4A01G359200	-	4A	632,150,159	632,155,404	0.80
TraesCS4A01G370500	ABC transporter G family member	4A	642,209,939	642,213,850	-0.94
TraesCS4A01G386400	Auxin repressed/dormancy associated protein	4A	663,986,105	663,987,373	0.59
TraesCS4A01G593300LC	3'(2'),5'-bisphosphate nucleotidase 1	4A	674,553,838	674,556,604	0.69
TraesCS4A01G401700	Cysteine synthase	4A	675,600,394	675,603,117	0.53
TraesCS4A01G599200LC	Zinc finger MYM-type protein 4	4A	677,402,029	677,403,093	1.26
TraesCS4A01G404800	arabinogalactan protein 5	4A	678,340,392	678,341,039	-0.78
TraesCS4A01G421900	Histone H3	4A	692,358,749	692,359,852	-0.43
TraesCS4A01G454800	Glutathione S-transferase	4A	718,848,586	718,850,423	0.52
TraesCS4A01G473900	F-box protein	4A	733,670,552	733,672,307	1.01
TraesCS4A01G485900	Beta-fructofuranosidase 1	4A	739,310,199	739,314,035	**-1.50**
TraesCS4A01G486600	disease resistance family protein / LRR family protein	4A	739,627,540	739,630,396	**-1.64**

[a] The genes model with LC are low confidence gene model and those without LC are High confidence gene models.

[b] Beta value is analogous to fold-change in slueth. A fold change of an absolute value of 1.5 or higher is considered differentially expressed and indicated in **bold**. Highly differentially expressed genes" correspond to those with beta > |5|. A positive fold-change means there was more expression in *ERA8* compared to WT. A negative fold-change means there was more expression in WT compared to *ERA8*. Highly differentially expressed genes" correspond to those with beta > |5|
